# Synthesis and properties of poly(*p*-phenylene vinylene) derivatives with hyperbranched structure and containing a nitro substituent

**DOI:** 10.1007/s00706-013-1051-2

**Published:** 2013-08-14

**Authors:** Ruyu Li, Yanjiao Mo, Rong Shi, Peng Li, Chengyu Li, Zhenjiang Wang, Xun Wang, Shengbiao Li

**Affiliations:** 1 College of Chemistry, Central China Normal University (CCNU), Wuhan, 430079 Hubei China; 2 Raffles Institution, 1 Raffles Institution Lane, Singapore, 575954 Singapore

**Keywords:** Nitro substituent, Hyperbranched, Poly(*p*-phenylene vinylene) derivatives, Synthesis, Properties

## Abstract

**Abstract:**

In order to improve efficiency, processability, and stability, two groups of novel poly(*p*-phenylene vinylene) (PPV) derivatives (P_1_–P_3_ and P_4_–P_6_) with hyperbranched structure and containing a nitro substituent were synthesized via a Gilch reaction in different monomer ratios. The properties of the polymers were investigated by using UV–Vis absorption, fluorescence spectroscopy, cyclic voltammetry, and thermogravimetric analysis. The result shows that the band gaps of the PPV derivatives with a nitro substituent were decreased and the polymers had higher molecular weights (10^6^), excellent solubility in common organic solvents, good film-forming ability, and better thermal stability. The polymers can be used as an efficient acceptor material in polymeric solar cells.

**Graphical abstract:**

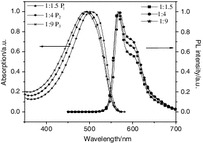

## Introduction

Since the discovery of electroluminescent poly(*p*-phenylene vinylene) (PPV) in 1990 [1], PPV continues to receive considerable interest for applications such as light-emitting diodes, field effect transistors, and photovoltaic devices owing to the polymer’s unique physical properties such as conductivity, electroluminescence, and solubility of selected derivatives [2–5]. PPV also encounters some problems, such as its tendency to aggregate, formation of excitation in the solid state leading to blue-green emission, fluorescence quenching, and the decrease in electroluminescence efficiency [6–8]. Although PPV is one of the best materials to be used as an efficient acceptor in polymeric solar cells (PSCs) because its structure causes low solubility and aggregation, the copolymers with other units which can overcome these problems have been investigated extensively.

Recently, hyperbranched polymers have drawn much attention and consideration owing to their ability to diminish the formation of interchain interactions owing to their high degree of branching, which can improve the solubility and thermal stability of PPV and reduce chain aggregation caused by fluorescence quenching. Considering these merits, we sought to synthesize a new kind of PPV which has hyperbranched structure [9–13].

Modified PPVs containing electron-withdrawing groups such as fluorine atoms, cyano groups, and heterocycles display high electron affinities (EA) and electron-transport properties as a result of increasing the polymer’s electron deficiency. The nitro moiety is a strong electron-withdrawing group, and inductive conjugation effects in nitrocellulose not only improve the nitro group’s electronic cloud density and increase the charge capacity, but also can enhance the electronic properties of conjugated polymers, increase the interaction between charges, and improve energy transfer and the quenching efficiency [14]. In order to reduce the band gap effectively, strong electron-withdrawing groups have been incorporated into the main skeleton to form a donor–acceptor (D–A) bridge.

Various modified Wessling reactions which originally use sulfur-based leaving groups have all been reported for the synthesis of a range of PPV derivatives [15–17]. This method has some drawbacks, such as the generation of toxic monomer during the solid-state elimination process, structural defects, and indefinable distribution of the molecular weights. The Gilch route is most widely used with long alkyl or alkoxy chains brought into the phenylene ring before polymerization to ensure the solubility [18, 19]. In this paper, we describe the synthesis and properties of a new family of hyperbranched copolymers. A novel “A_2_ + B_3_” approach based on Gilch polycondensation is used for the polymer synthesis.

In this publication, we report the synthesis and characterization of two groups of novel PPV derivatives (P_1_–P_3_ and P_4_–P_6_) with hyperbranched structure and containing a nitro substituent. Both groups of polymers exhibit a molecular weight of approximately 10^6^, better solubility, a wide range of spectral absorption, and good thermal stability. These characteristics explain why the polymer is a good photoelectric material.

## Results and discussion

The detailed syntheses of the two groups of hyperbranched polymers are outlined in Scheme 1. The polymerization was done under different monomer feed ratios in tetrahydrofuran (THF) with freshly prepared potassium *tert*-butoxide. Polymers P_1_, P_2_, and P_3_ were obtained by reacting monomer **A** and **4** in molar ratios of 1.5:1, 4:1, and 9:1, respectively. Polymers P_4_, P_5_, and P_6_ were obtained by reacting monomer **A** and **B** in molar ratios of 3:1, 6:1, and 9:1, respectively. All polymers are soluble in common organic solvents, such as toluene, THF, chloroform, and methylene chloride. The molecular structures of the polymers were characterized with high-resolution NMR spectroscopy. ^1^H NMR revealed that the chemical shifts of the three symmetrical –CH_2_Br groups on the aryl of monomer **4** and **B** were about 4.45 ppm; those peaks disappeared and new vinylic proton peaks (at 7.45 ppm) and aromatic proton peaks appeared in the ^1^H NMR spectra of the polymers, suggesting that the polymerization was complete.

**Figure:**
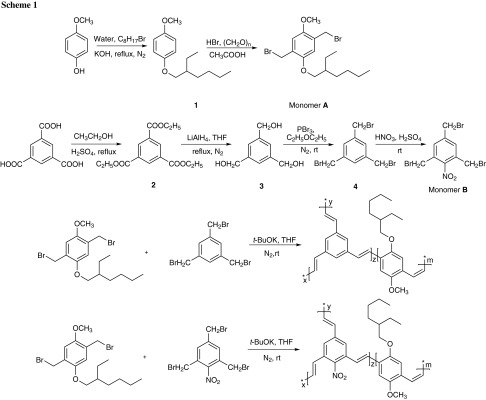

The number-average molecular weight (*M*_n_) and the weight-average molecular weight (*M*_w_) were determined by gel permeation chromatography (GPC) against standard polystyrene. The molecular weight values were merely estimates because of the differences in hydrodynamic radius between the hyperbranched polymers and the polystyrene standard. As shown in Table 1, the hyperbranched PPV derivatives prepared via Gilch reaction exhibit high molecular weights.

**Table 1:** GPC of P_1_–P_6_

Polymer	*M*_n_ × 10^6^	*M*_w_ × 10^6^	PDI
P_1_	1.76	3.36	1.91
P_2_	1.27	2.45	1.93
P_3_	2.30	3.75	1.63
P_4_	1.90	3.88	2.05
P_5_	3.09	5.90	1.91
P_6_	1.50	3.77	2.59

*PDI* polydispersity index

### Thermal properties

**Fig. 1:**
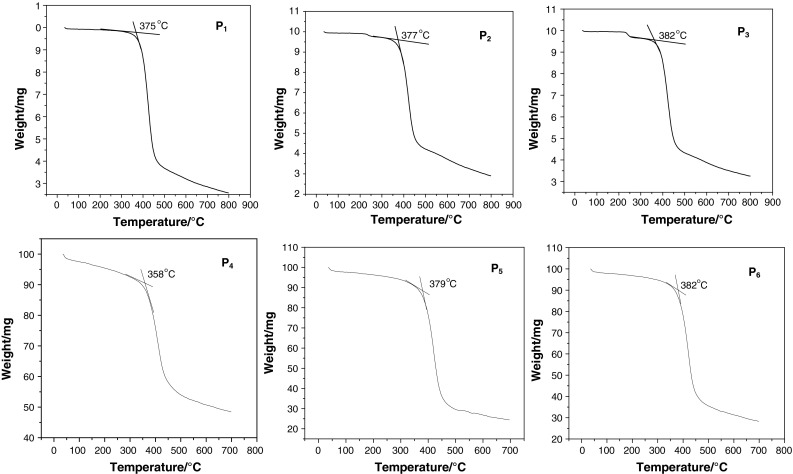
TGA of P_1_–P_6_

The thermal properties of the hyperbranched polymers were investigated using thermogravimetric analysis (TGA). The polymers exhibited high thermal stability and onset of weight loss temperatures of 375, 377, 382 and 358, 379, 382 °C for P_1_, P_2_, P_3_ and P_4_, P_5_, P_6_, respectively, as shown in the TGA curves in Fig. 1. They were much higher than those for the linear MEH-PPV. The high thermal stability of these polymers could be of benefit to increase the stability of the PSC, which prevents morphological change, deformation, and degradation of the active layer [20].

### Electrochemical properties

Cyclic voltammetry (CV) was employed to investigate the electrochemical properties of the six copolymers. The oxidation and reduction potentials revealed in cyclic voltammograms indicate the highest occupied molecular orbital (HOMO) and lowest unoccupied molecular orbital (LUMO) levels, which correspond to ionization potentials (IP) and EA, respectively [21]. The CV of polymer films cast from THF solution onto glassy carbon plate working electrodes, in 0.1 M tetrabutylammonium hexafluorophosphate (TBAPF_6_) in anhydrous acetonitrile as supporting electrolyte, were measured at a scanning rate of 10 mV/s.

The HOMO and LUMO energy levels of polymers were calculated and the onset potentials for oxidation and reduction are shown in Table 2. Firstly, without a nitro substituent (i.e., P_1_, P_2_, and P_3_), the onset potentials for oxidation (*E*_onset_^ox^) were 1.39, 1.18, and 1.02 V and the onset potentials for reduction (*E*_onset_^red^) were −1.67, −1.84, and −1.99 V. Secondly, with a nitro substituent (i.e., P_4_, P_5_, and P_6_), the onset potentials for oxidation (*E*_onset_^ox^) were 0.99, 0.97, and 0.91 V and the onset potentials for reduction (*E*_onset_^red^) were −1.80, −1.86, and −1.90 V.

**Table 2:** Electrochemical properties of P_1_–P_6_

Polymer	*E*_g_/eV	*E*_HOMO_/eV	*E*_LUMO_/eV
P_1_	3.06	−5.79	−2.73
P_2_	2.98	−5.58	−2.59
P_3_	3.01	−5.42	−2.41
P_4_	2.79	−5.39	−2.60
P_5_	2.82	−5.37	−2.55
P_6_	2.81	−5.31	−2.50

$$ E_{\text{HOMO}} = \, - e \, \left( {E_{\text{ox}} + { 4}. 4} \right) \, \left( {\text{eV}} \right) $$

$$ E_{\text{LUMO}} = \, - e \, \left( {E_{\text{red}} + { 4}. 4} \right) \, \left( {\text{eV}} \right) $$

The HOMO energy levels of the two groups of polymer were gradually reduced. The reason is possibly that the ratio of monomer **A** in these polymers increases little by little. The result indicates that hole-injection and transporting properties have been improved. The result implies that the electron-rich alkoxy groups affect the electronic properties of the conjugated copolymer chains significantly. Comparison of the two groups of polymers shows that the introduction of a nitro group in the hyperbranched PPVs not only increased the HOMO energy level, but also made the band gap narrower.

### Photophysical properties

**Fig. 2:**
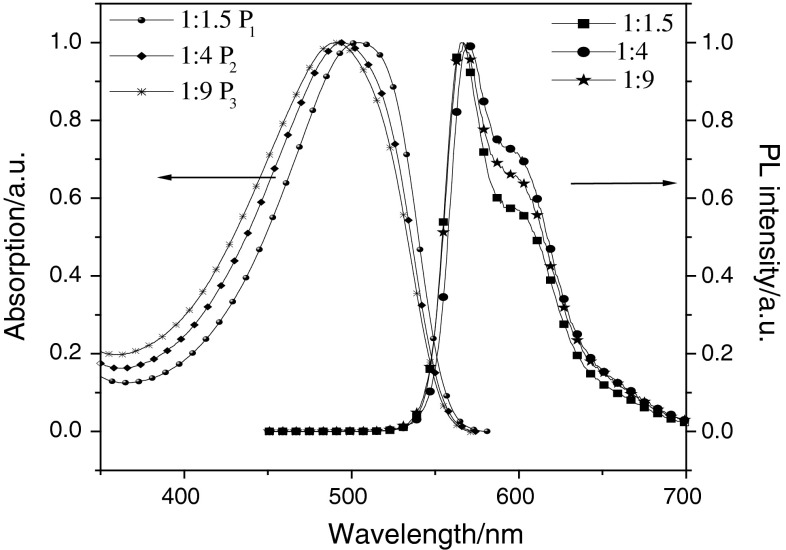
UV–Vis absorption spectra and PL spectra of P_1_, P_2_, and P_3_ in CHCl_3_ (1 × 10^−6^ g/cm^3^)

**Fig. 3:**
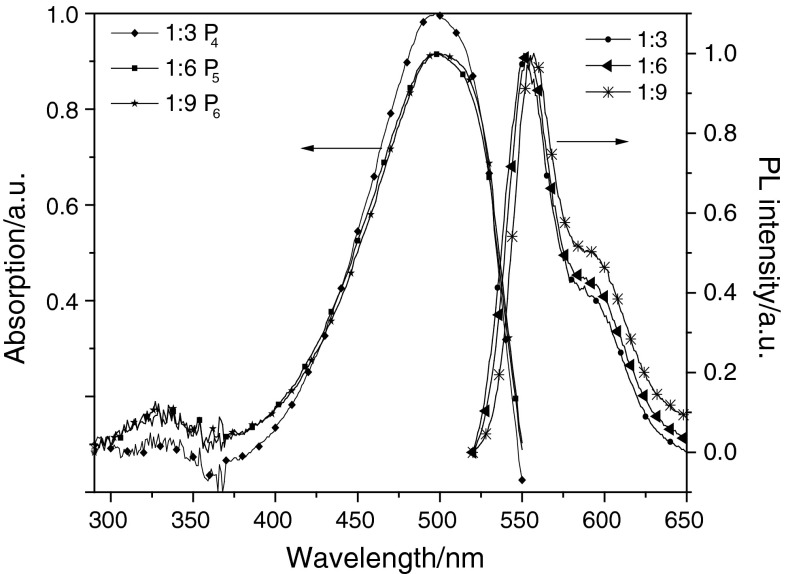
UV–Vis absorption spectra and PL spectra of P_4_, P_5_, and P_6_ in THF (1 × 10^−6^ g/cm^3^)

**Fig. 4:**
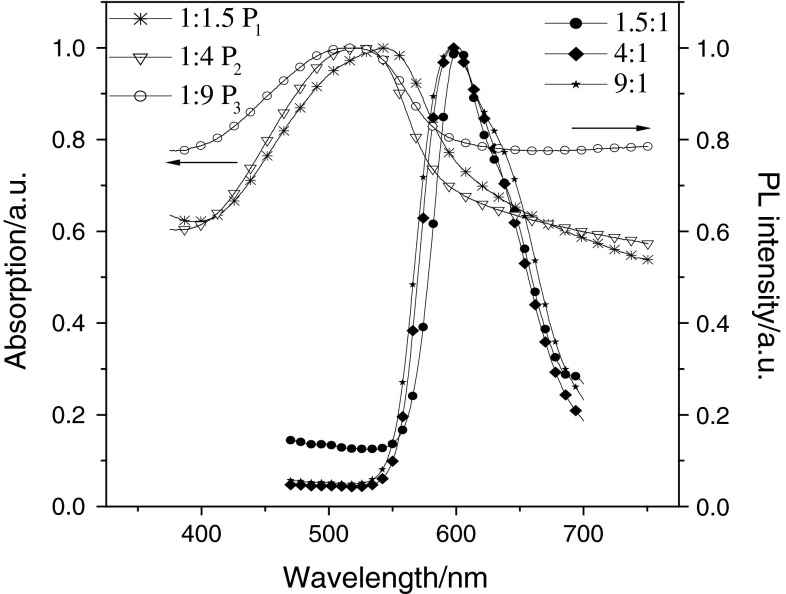
UV–Vis absorption spectra and PL spectra of P_1_, P_2_, and P_3_ in the film

**Fig. 5:**
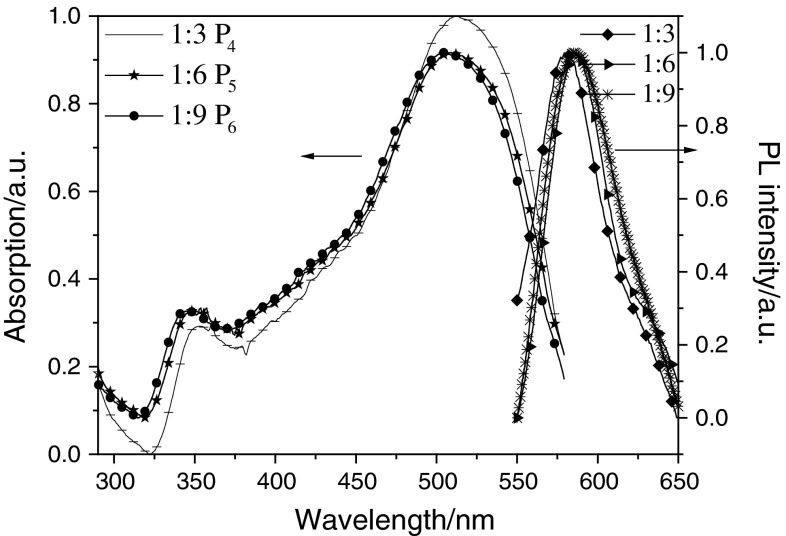
UV–Vis absorption spectra and PL spectra of P_4_, P_5_, and P_6_ in the film

The photophysical characteristics of the polymers were investigated in solution and the thin film. The UV–Vis absorption and photoluminescence (PL) spectra of P_1_–P_3_ and P_4_–P_6_ in solution are shown in Figs. 2 and 3. Those for P_1_–P_3_ and P_4_–P_6_ in the film are shown in Figs. 4 and 5. The UV–Vis absorption maximum and PL emission maximum are summarized in Table 3. According to the spectra in Fig. 2, polymers P_1_–P_3_ exhibit only major absorption at 503, 493, and 490 nm in solution. In Fig. 3, P_4_–P_6_ show maximum absorptions at 497 nm in solution, and P_4_–P_6_ exhibit other major absorptions at 329, 333, and 335 nm in solution. Both groups of polymers absorb at around 500 nm, which is attributed to the transitions of main-chain π-systems of the polymer. The absorptions in the short wavelength region of the second group of polymers originate from the nitro substituent. The maximum absorption peak of polymers with a nitro substituent is sharper than that of polymers without this substituent. This is the reason why introducing the strong electron-withdrawing nitro group is good for the polymer π–π* interaction. The thin film absorption spectra show an obvious red shift relative to those in solution. According to the data given in Table 3, this red shift of the six polymers P_1_–P_6_ is 38, 29.5, 27, 8, 8, and 13 nm, respectively. The PL spectra were recorded with an excitation wavelength corresponding to the absorption maximum wavelength of the polymer. Emission peaks of P_1_, P_2_, and P_3_ in chloroform solution were all 567 nm, and in thin film were 602, 596, and 596 nm, respectively. Meanwhile, the emission peaks of P_4_, P_5_, and P_6_ in THF solution were all 553 nm, and in thin film were 581, 586, and 583 nm, respectively. This red shift could be explained by the enhancement of the interaction in the solid states.

**Table 3:** Optical properties of P_1_–P_6_

Polymer	UV–Vis		PL	
	*λ*_max_ solution/nm	*λ*_max_ film/nm	*λ*_max_ solution/nm	*λ*_max_ film/nm
P_1_	503	542	567	602
P_2_	493	523	567	596
P_3_	490	517	567	596
P_4_	329, 497	338, 505	553	581
P_5_	333, 497	341, 505	553	586
P_6_	335, 497	350, 510	553	583

## Conclusion

We have successfully synthesized two groups of novel PPV derivatives (P_1_–P_3_ and P_4_–P_6_) with hyperbranched structure and containing a nitro substituent via the Gilch reaction. The introduction of trisubstituted benzene resulted in the twisting of the polymer backbone and thus decreased coplanarity. By introducing trisubstituted benzene with a nitro substituent, it increased the absorption range and lowered the polymer band gap. Poly-(1,3,5-tris(bromomethyl)benzene co 1,4-Bis(bromomethyl)-2-methoxy-5-(2’-ethyl)hexyloxybenzene phenylene vinylenene) (PTV-*co*-MEHPV) and Poly-(2-Nitro-l,3,5-tris(bromomethyl)benzene co 1,4-Bis(bromomethyl)-2-methoxy-5-(2’-ethyl)hexyloxybenzene phenylene vinylenene) (PTNV-*co*-MEHPV) have good solubility in common organic solvents. The number-average molecular weight and weight-average molecular weight are also large. All polymers we synthesized are red-light-emitting materials. The polymers can be used as an efficient acceptor material in PSC.

## Experimental

^1^H NMR spectra of the compounds and polymers were recorded on a 400 MHz Varian Mercury NMR spectrometer in CDCl_3_ at room temperature, containing a small amount of TMS as internal standard. The molecular weights of the polymers were determined by GPC using a Perkin-Elmer series 200 apparatus in THF with polystyrene as standards. The flow rate of THF was maintained as 1.0 cm^3^/min at 40 °C. The thermal stability of the polymers was determined using a STA409 PC at a heating rate of 10 °C/min in a nitrogen atmosphere. The absorption and emission studies were done using a U-3310 UV–Vis spectrophotometer and a RF-5301P spectrofluorometer. The electrochemical measurements were performed using CV-50 W at a constant scan rate of 100 mV/s, with 0.1 M TBAPF_6_ solution in acetonitrile as the supporting electrolyte. A platinum wire and an Ag/AgNO_3_ electrode were used as the counter electrode and reference electrode, respectively. The solution spectra were recorded in THF and CHCl_3_ solutions, whereas the solid-state spectra were obtained from polymer thin films prepared by spin-coating the 10^−3^ mol/dm^3^ THF and CHCl_3_ solutions at 1,500 rpm on glass substrates.

Precursors **1**–**4** and monomers **A** and **B** were prepared according to literature procedures [22–24].

### Polymer synthesis

A typical polymerization procedure for the preparation of the new polymers is described as follows. A solution of 8.4 cm^3^ of potassium *tert*-butoxide (2.0 M THF solution, 16.8 mmol) was slowly added over 1 h to a stirred solution of the two monomers at the chosen molar ratio (see below) in 25 cm^3^ dry THF that was cooled to 0 °C and under N_2_ atmosphere. The mixture was stirred at room temperature for 20 h. The polymerization solution was poured into 500 cm^3^ methanol. A crude polymer precipitated out, which was redissolved in chloroform and reprecipitated from methanol and then extracted in a Soxhlet apparatus with methanol to remove the impurities and oligomers. After drying filtration in vacuum, a bright red solid was obtained. In this paper, we adopt different ratios of starting monomers for the synthesis of the two groups of polymers. The proportions for P_1_–P_3_ are monomer **A** (0.2, 0.2, 0.1 mmol) and **4** (0.3, 0.8, 0.9 mmol); those for P_4_–P_6_ are monomer **A** (0.2, 0.2, 0.1 mmol) and monomer **B** (0.6, 1.2, 0.9 mmol). ^1^H NMR of P_1_ and P_4_ (400 MHz, CDCl_3_): *δ* = 7.54–7.09 (m, Ar–H), 4.14–3.85 (m, –OCH_2_–, –OCH_3_), 1.95–1.94 (m, –OCH_2_CH), 1.55–1.25 (m, –CH_2_), 0.89 (t, –CH_3_) ppm; ^13^C NMR of P_1_ (100 MHz, CDCl_3_): *δ* = 10.09, 13.99, 22.42, 30.50, 38.12, 39.56, 55.79, 65.29, 109.01, 124.40, 134.56, 144.22 ppm; ^13^C NMR of P_4_ (100 MHz, CDCl_3_): *δ* = 8.85, 17.44, 24.45, 32.22, 40.01, 51.73, 61.73, 110.76, 122.41, 137.91, 143.84, 145.51, 148.69 ppm.

## References

[1] Burroughes JH, Bradley DDC, Brown AR, Marks RN, Mackay K, Friend RH, Burns PL, Holmes AB (1990). Nature.

[2] Colladet K, Fourier S, Cleij TJ, Lutsen L, Gelan J, Vanderzande D, Huong Nguyen L, Neugebauer H, Sariciftci S, Aguirre A, Janssen G, Goovaerts E (2007). Macromolecules.

[3] Cho NS, Park JH, Lee SK, Lee J, Shim HK, Park MJ, Hwang DH, Jung BJ (2006). Macromolecules.

[4] Thompson BC, Kim YG, Reynolds JR (2005). Macromolecules.

[5] Sun XB, Zhou YH, Wu WC, Liu YQ, Tian WJ, Yu G, Qiu WF, Chen SY, Zhu DB (2006). J Phys Chem B.

[6] Chu HY, Hwang DH, Do LM, Chang JH, Shim HK, Holmes AB (1999). Synth Met.

[7] Hsieh BR, Yu Y, Forsythe EW, Schaaf GM, Feld WA (1998). J Am Chem Soc.

[8] Peng Z, Zhang J, Xu B (1999). Macromolecules.

[9] Wooley KL, Frechet JMJ, Hawker CJ (1994). Polymer.

[10] Shirota Y, Kuwabara Y, Inada H, Wakimoto T, Nakada H, Yonamoto Y, Kawai S, Imai K (1994). Appl Phys Lett.

[11] Wang KL, Huang ST, Hsieh LG, Huang GS (2008). Polymer.

[12] Wang KL, Kakimoto M, Jikei M (2005). High Perform Polym.

[13] Wan WM, Pan CY (2008). Macromolecules.

[14] Babudri F, Farinola GM, Naso F, Ragni R (2007) Chem Commun 1003 10.1039/b611336b 17325792

[15] Louwet F, Vanderzande D, Gelan J, Mullens J (1995). Macromolecules.

[16] Lutsen L, Adriaensens P, Becker H, Van Breemen AJ, Vanderzande D, Gelan J (1999). Macromolecules.

[17] Son S, Dodabalapur A, Lovinger AJ, Galvin ME (1995). Science.

[18] Papadimitrakopoulos F, Konstadinidis K, Miller TM, Opila R, Chandross EA, Galvin ME (1994). Chem Mater.

[19] Braun D, Heeger AJ (1991). Appl Phys Lett.

[20] Tokito S, Tanaka H, Noda K, Okada A, Taga Y (1997). Appl Phys Lett.

[21] Tsai LR, Chen Y (2008). Macromolecules.

[22] Neef CJ, Ferraris JP (2000). Macromolecules.

[23] Díez-Barra E, García-Martínez JC, Merino S, del Rey R, Rodríguez-López J, Sánchez-Verdú P, Tejeda J (2001). J Org Chem.

[24] West AP, Smyth N, Kraml CM, Ho DM, Pascal RA (1993). J Org Chem.

